# AI in variant analysis: fast track to genetic diagnoses

**DOI:** 10.1007/s00439-026-02847-0

**Published:** 2026-06-26

**Authors:** Elizabeth J. Wilk, Sasha Taluri, Timothy C. Howton, Anthony B. Crumley, Michal Mrug, Brittany N. Lasseigne

**Affiliations:** 1 The Department of Cell, Developmental and Integrative Biology, Heersink School of Medicine, The University of Alabama at Birmingham, Birmingham, AL USA; 2 The Department of Medicine, Heersink School of Medicine, The University of Alabama at Birmingham, Birmingham, AL USA; 3 Department of Veterans Affairs Medical Center, Birmingham, AL USA

## Abstract

While falling costs have expanded access to genomic sequencing, clinical utility is frequently hindered by the challenge of interpreting complex genetic data. Variant analysis for rare disease patients especially requires significant time and expertise, creating a bottleneck that delays diagnostics. Although advances in genetic variant classification have improved diagnostic precision, they have also increased the identification of variants of uncertain significance (VUSs), widening the interpretation gap between data generation and clinical actionability. The high prevalence of VUSs can lead to false reassurance or psychological distress by misinterpretting inconclusive results. We propose that artificial intelligence (AI) is a critical clinical decision-support tool for bridging this gap, offering a scalable framework to optimize variant interpretation and shorten the diagnostic odyssey. While reclassification ultimately requires biological evidence that AI cannot replace, these tools serve as essential aggregators and prioritizers, especially as guidelines transition toward the upcoming quantitative ACMG v4 framework. We advocate integrating AI throughout the genetic diagnostic workflow–from initial phenotyping to variant prioritization–to facilitate data-driven, personalized treatment. We outline current AI-assisted approaches and discuss anticipated challenges in this pursuit, such as privacy, training data bias and quality, model explainability, and the necessity of a total product life cycle for validation. To address these challenges, we provide recommendations for "human-in-the-loop" design and intuitive workflow integration to ensure AI tools meet the highest standards of precision, reproducibility, and transparency to maximize adoption. By standardizing AI across the variant analysis pipeline, we can fast-track the path to genetic diagnoses, effectively bridging the interpretation gap and enabling rapid delivery of personalized medical interventions.

## Introduction

**Fig. 1:**
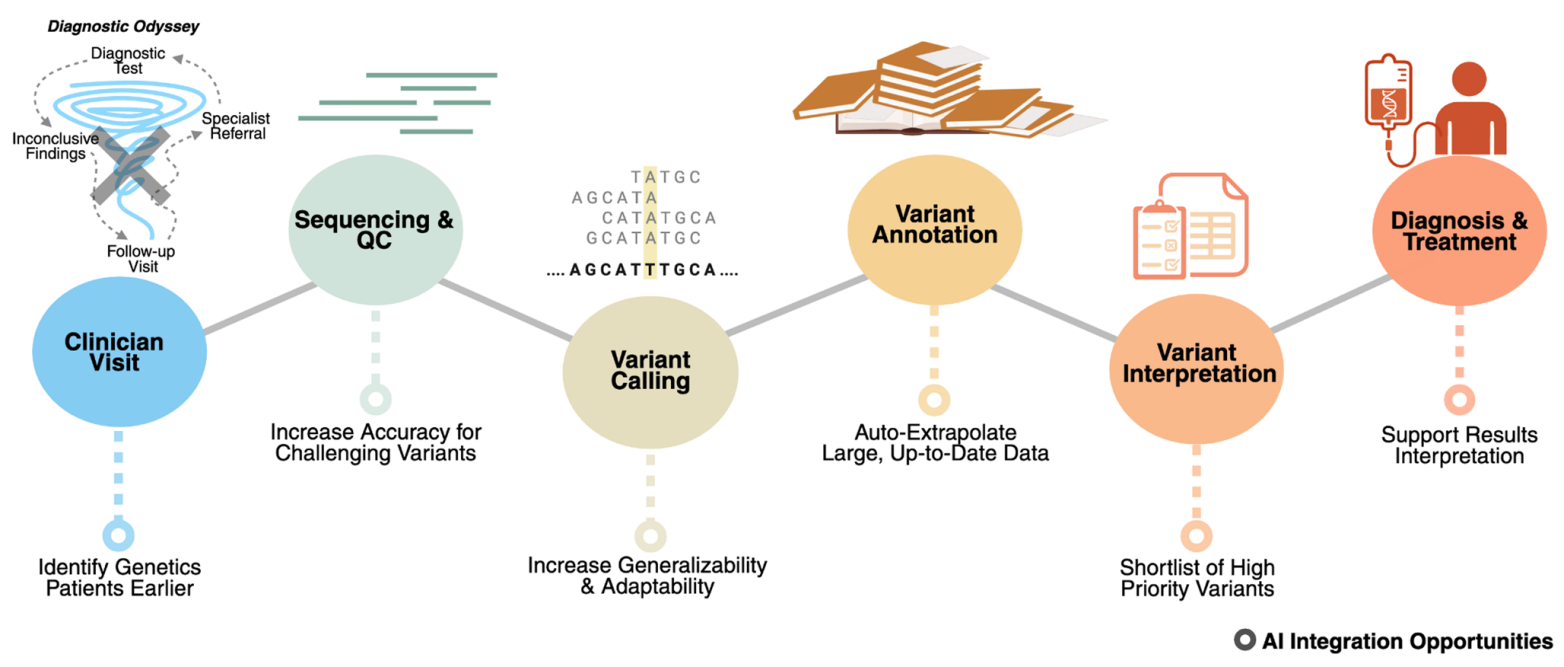
Flowchart demonstrates targeted AI opportunities at each step of the genetic diagnostic workflow. Created in BioRender. https://BioRender.com/6xxko8q

The average time to receive a genetic diagnosis for a rare disease across high-income countries ranges from 4 to 19 years (Faye et al. 2024; Phillips et al. 2024), resulting in $86,000 to $516,000 in avoidable costs per patient (Lewin Group 2023). Current practices force patients with genetic diseases into a "diagnostic odyssey," subjecting them to rounds of unnecessary clinic visits, procedures, and medications (Fig. 1). This process closes or narrows their window of intervention, enabling disease progression and long-term disease damage. High-throughput genetic testing, critical for addressing the diagnostic odyssey, has become widely accessible and cost-effective (Kris and Wetterstrand 2019). Even when paid out of pocket, sequencing costs are a fraction of the overall odyssey’s costs.

Approximately 30 million Americans have a genetic disease (~ 1 in 10) (Wan et al. 2023; Lewin Group 2023). Therefore, early disease identification and therapeutic intervention should be the norm. However, physicians report limitations in their genetics training (Rasouly et al. 2023; Peabody et al. 2015; Kneifati-Hayek et al. 2024), and many express reduced interest in genetic screening due to the rarity of genetic conditions (Pasquier et al. 2022; Wan et al. 2023).

Variant analysis—the rate-limiting step in genetic testing (Tagliafico et al. 2018)—classifies variants by pathogenicity to guide clinical decision-making. Inaccurate interpretation at this stage fundamentally alters patient management, preventing the use of targeted therapies, initiating surveillance, or performing preventive procedures (Agaoglu et al. 2022). These errors also extend to the family, obscuring the need for cascade screening or preimplantation genetic diagnosis (McNeill 2022). Consequently, misinterpreted variants contribute to avoidable morbidity and mortality through missed preventative interventions, while simultaneously inflicting psychological harm via false reassurance or unnecessary anxiety (Campeau 2022). The standard of care uses American College of Medical Genetics and Genomics (ACMG)/Association for Molecular Pathology (AMP) and/or European Society of Human Genetics (ESHG) guidelines (Richards et al. 2015; Houge et al. 2022) for variant interpretation, but the process as a whole remains labor-intensive and relies heavily on experts. Results can be inconsistent and often yield variants that lack sufficient evidence to be classified as benign or pathogenic (Zukin et al. 2023; Agaoglu et al. 2022; Lin et al. 2023), complicating patient care. However, automation that leverages all available clinical, molecular, and population data in a standardized, reproducible manner could help reduce these issues. Artificial intelligence (AI), tools with ”human-like reasoning” built from a variety of machine learning (ML) models and/or large language models (LLMs) (reviewed in Russell and Norvig 2021; Janiesch et al. 2021; Koteluk et al. 2021; Nichols et al. 2019), can optimize labor- and knowledge-intensive steps throughout the genetic testing process.

In this perspective (Table 1), we highlight opportunities, challenges, and recommendations for incorporating AI into variant analysis to support clinical genetic testing and research.

*Descriptive caption*: Flowchart illustrating AI integration opportunities in genetic diagnostics, progressing through five stages: Clinician Visit, Sequencing & QC, Variant Calling, Variant Annotation, Variant Interpretation, and Diagnosis & Treatment. Each stage is represented by colored circles with icons and brief descriptions highlighting improvements such as increased accuracy, generalizability, data extrapolation, variant prioritization, and support for results interpretation.

## Approach to variant analysis

AI is emerging at a time when clinical genetics faces its greatest gap between knowledge and practice.

To prevent the diagnostic odyssey (Fig. 1), physicians must first recognize patients who would benefit from genetic testing. Genetic diseases typically present with a constellation of symptoms (e.g., dysmorphism, early-onset, and/or multi-system involvement); therefore, AI could assist in determining when genetic testing may be appropriate (e.g., FACE2GENE (Gurovich et al. 2019). For instance, AI integrations in electronic health records (EHRs) could detect potential patients, even those with subtle clinical presentations (Ye et al. 2024; Yang et al. 2022). AI can also support physician training in genetic results interpretation through adaptive educational modules (Hajek et al. 2022).

After sequencing, variant analysis processes the data in four key steps: variant calling, annotation, prioritization, and interpretation (Fig. 1). AI/ML tools have already streamlined variant calling by reducing manual filtering and improving scalability. Examples of this include Google’s DeepVariant (Poplin et al. 2018), DNAscope (Freed et al. 2022; Hu et al. 2025), DeepTrio (Hu et al. 2022), Clair3 (Su et al. 2022), Medaka (Nagy et al. 2026), and HELLO (Ramachandran et al. 2021). These tools offer speed and generalizability across sequencing platforms (Abdelwahab et al. 2023; Brand et al. 2024; Abdelwahab and Torkamaneh 2025).

Following variant identification, variant annotation contextualizes a patient’s variants using available functional and clinical data. This step requires synthesizing sequence data, conservation, population frequency, and variant effect (e.g., missense, nonsense, frameshift) across diverse databases. LLMs, a subtype of AI models that process and generate human language (Cloud4C 2024), excel at automating this process. Mining resources like ClinVar and gnomAD (i.e., large databases of patient variants) have been assessed in the context of their genetic sequences to predict a variant’s consequences on the RNA (e.g., SpliceAI and Evo2) or protein’s primary structure (e.g., AlphaMissense)(Tordai et al. 2024; Brixi et al. 2026). Other ML models have enhanced variant annotation through feature-based learning (e.g., REVEL, CADD, PrimateAI-3D)(Ioannidis et al. 2016; Kircher et al. 2014; Gao et al. 2023).

End-to-end variant analysis pipelines, including AI-MARRVEL (Mao et al. 2024), Qiagen’s Franklin (QIAGEN Digital Insights 2025), Illumina’s Emedgene (Meng et al. 2023), Nostos Genomics, and VarSome Clinical (Kopanos et al. 2019), have already automated many of the steps required for variant interpretation and prioritization. These tools use large, aggregated variant annotation data and *in silico* predictions to automate ACMG/AMP variant classification by applying a Bayesian point-based system (Tavtigian et al. 2020) and using context-aware weight adjustments. For example, while certain criteria — such as PM2 (absence in control populations) — are relatively straightforward to automate, others rely on subjective qualitative definitions, such as PM1 (located in a "mutational hot spot") (Richards et al. 2015). Platforms like VarSome overcome this challenge by applying statistically calibrated rule strengths and thresholds, enabling objective, semi-automated evaluation while maintaining a transparent "human-in-the-loop" framework for final clinical review (Saphetor 2026). Furthermore, upcoming ACMG/AMP v4 guidelines will transition to a more quantitative, Bayesian, points-based framework with variants of uncertain significance (VUSs) sub-classifications of “High”, “Mid”, and “Low” tiers, stratifying highly suspect variants with limited data for further investigation (Biesecker et al. 2026).

While AI-augmented tools and pipelines have significantly improved variant analysis timelines and standardization, the paths to reaching diagnosis are often stunted by VUSs. Currently, variants require ≥ 90% certainty of clinical relevance (Richards et al. 2015) to classify a variant as benign, likely benign, likely pathogenic, or pathogenic. As such, VUSs remain the most common variant classification, accounting for ~ 35–37% of variants associated with rare diseases and cancer (Balmaña et al. 2016; Zawar et al. 2025; Fowler and Rehm 2024). VUS reclassification typically requires the generation of more biological evidence — functional assays, segregation analysis, new case observations, etc — which AI cannot replace. However, AI can indirectly reduce VUSs by curating evidence and improving ACMG/AMP criteria application consistency. Furthermore, computational models can extrapolate from existing, experimentally characterized training data to improve our contextual understanding of complex genetic alterations, such as non-coding, splice, low-penetrance, or hypomorphic variants. While restricted by available data, these tools provide robust, computationally derived supporting evidence, rather than serving as standalone replacements for experimental validation. For example, DYNA, a disease-specific LLM, compares context-specific networks to score the pathogenicity of coding and non-coding variants (Zhan and Zhang 2024). In a study of > 17k cardiomyopathy VUSs from ClinVar, DYNA reclassified ~ 9% as pathogenic, likely pathogenic, benign, or likely benign (Zhan and Zhang 2024). Another promising approach to improving classification supporting evidence is to estimate penetrance. In rare diseases, small cohorts make it difficult, or even impossible, to calculate penetrance using traditional methods. However, efforts are being made; for example, Forrest et al. (2025) recently developed disease-specific ML models to calculate disease probability and penetrance using EHR and genetic data.

AI-assisted variant analysis can clarify genetic test results (e.g., AI-enabled ACMG scoring within EHRs and clinical trial eligibility screening (Jin et al. 2024), enabling clinicians to weigh genomic evidence alongside clinical findings. With data-driven rationales to support clinical diagnostics, clinicians are better equipped to make more efficient and accurate decisions. Clinicians can thereby reduce trial-and-error prescribing by linking variants to targeted therapies and trials. Ultimately, AI assistance will increase genetic screening rates, preventing delays in care.

## Challenges and recommendations

Integrating AI into clinical genetics shows great promise, but we expect challenges ahead (Table 1).

**Table 1:** AI integration challenges and recommendations in clinical genetics

Challenge	Recommendation	Impact
Privacy & Safety	Adhere to *GDPR, *HIPAA, *ISO/IEC, and *GINA; use secure data handling practices	Protect sensitive information and maintain patient trust
Data Quality & Bias	Use high-quality, representative datasets; avoid “big data hubris”	Reduce bias, improve prediction accuracy, and ensure fairness
Model Transparency	Incorporate explainable AI (XAI) methods; ensure models are auditable	Improve trust, interpretability, and ethical accountability
Validation & Life Cycle	Implement post-market testing and total product life cycle monitoring	Ensure the ongoing efficacy and safety of AI tools

The table summarizes potential challenges using our proposed AI−assisted approach, along with recommended solutions and their expected clinical impact. *General Data Protection Regulation (GDPR), *Health Insurance Portability and Accountability Act (HIPAA), *International Organization for Standardization (ISO), *International Electrotechnical Commission (IEC), *Genetic Information Nondiscrimination Act of 2008 (GINA)

Meaningful impact necessitates widespread adoption and ethical, rigorously tested tools. Despite its promise, global public opinion toward AI remains cautious (Poushter et al. 2025), especially for healthcare (Reis et al. 2024). Because many physicians report limitations in their genetics training (Rasouly et al. 2023; Kneifati-Hayek et al. 2024; Mrug et al. 2021), we advocate for increasing clinician and patient involvement as collaborators throughout the development process to ensure relevance and interface usability (Erikson 2018). For example, including target community members as testers optimizes platforms to maximize intuitive, user-friendly interfaces while ensuring tools are seamlessly integrated into existing clinical workflows and EHR systems. By involving clinicians and patients and fostering shared understanding, researchers can build the transparency required to empower users and sustain long-term provider trust (Tomašev et al. 2020; McCoy et al. 2025).

Another primary concern by clinicians regarding AI use in variant interpretation is medical malpractice liability (Eltorai et al. 2024; Abdelwanis et al. 2026). Because AI serves as a decision-support tool, rather than an autonomous diagnostician, the legal responsibility for acting on a misclassified variant ultimately falls on the human provider. This risk can be mitigated using two critical safeguards: regulatory certification and Explainable AI (XAI) (Table 1). Selecting platforms with clinical certifications, such as a CE-IVDR Class C designation in Europe, helps shield providers by ensuring the software has undergone rigorous third-party conformity assessments for safety, analytical performance, and continuous quality management. For example, VarSome is CE-IVDR Class C certified(Saphetor 2025), while others are labeled for “research only use,” i.e., non-diagnostic use (e.g., Illumina’s Emedgene) (illumina 2024).

XAI returns information to the user regarding how and why decisions were made with the AI, empowering clinicians with the information they need to critically assess the software’s output. ML/AI models offer powerful capabilities for streamlining variant analysis by integrating multimodal data (e.g., genetic sequences, EHRs, biomedical knowledge graphs, and large-scale text mining) but often at the cost of interpretability, with many functioning as a “black box” (Gosiewska et al. 2021; Riuz, 2018). To ensure fairness and accuracy, especially in clinical contexts, models must be auditable and explainable. An auditable model acts as a “glass box,” where processes can be systematically examined and traced (e.g., by logging decision logic (Sina Gräupner et al. 2023) and data sources used as evidence (Mercurio et al. 2022; Meng et al. 2023; Allot et al. 2023; https://www.nostos-Genomics.com 2025b; May et al. 2022; Sina Gräupner et al. 2023). XAI techniques further enable users to dissect models and their predictions to assess the influence of individual features. Numerous XAI approaches are currently available — even for complex LLMs — despite their scale of parameters and training (Zhao et al. 2024a, b; Chen et al. 2024; Peng et al. 2025). This transparency and explainability is especially critical in healthcare to mitigate the risk of malpractice liability. Some AI-assisted variant analyses and workflows already incorporate XAI methods, such as scoring and ranking the importance of features that drive their predictions (Meng et al. 2023; Forrest et al. 2025; https://www.nostos-Genomics.com 2025a) (Lundberg and Lee 2017).

Genetic data has historically raised significant legal, ethical, and privacy concerns due to its uniquely identifiable nature. Using this data with AI raises additional concerns; therefore, it is vital that training data and software comply with national/international laws and standards (Genetic Information Nondiscrimination Act of 2008 (GINA) 2008; Ruiz, 2018; European Union 2016; Office for Civil Rights (OCR), n.d.; International Organization for Standardization, International Electrotechnical Commission 2022; Sokhansanj and Rosen 2025). Models for variant analysis should also adhere to established clinical standards from reputable organizations, such as the Human Genome Variation Society (HGVS) (den Dunnen et al. 2016), ACMG, AMP, CAP (Richards et al. 2015), and ESHG (Houge et al. 2022, 2024).

A major shortcoming of many AI tools stems from the data they are trained on. Overreliance on large, uncurated datasets can introduce bias, inaccuracies, and outdated information, leading to large errors in predictions (Lazer et al. 2014; Ross and Swetlitz 2018; Xing et al. 2025; Fieldhouse 2025; Kessler et al. 2016) and AI “hallucinations” (Beutel et al. 2023). Instead, datasets should be reliable and representative of the affected patient population (Tomašev et al. 2020; Nakayama et al. 2022; Daneshjou et al. 2022; Delgado et al. 2022; Center for Devices and Radiological Health 2025a; McCoy et al. 2025; Annual Report 2023, 2023). This is especially critical in biomedical applications, where underrepresentation can perpetuate disparities (Delgado et al. 2022; Daneshjou et al. 2022; Nakayama et al. 2022; Dastin 2018; Larson et al. 2016; Diaz et al. 2018; Obermeyer et al. 2019).

A promising solution to this is implementing retrieval-augmented generation (RAG) systems (curated knowledge bases), which have already aided biomedical applications and reduced AI hallucinations (Leiser et al. 2025; Lee et al. 2024). As variant classifications can change rapidly, relying on static LLM training data introduces a “lagging indicator” that is potentially hazardous for clinical diagnostics (e.g., VarSome re-annotates variants on a daily basis)(Saphetor 2026). RAG systems offer a more cost-effective and flexible alternative in these scenarios by updating the retrieval index of stored information instead of repeatedly retraining the model (Shen et al. 2025; Chatelain 2025). This mutability allows for the dynamic scaling and re-ranking of information, prioritizing expertly-curated databases over outdated or less reliable sources (Dhingra et al. 2022; Alexander 2026; P. Zhao et al. 2024a, b). However, the initial setup, infrastructure integration, and ongoing maintenance of these systems are costly investments (Wong et al. 2026; McCoy et al. 2025). While AI has the potential to reduce overall healthcare costs by improving diagnostic accuracy and optimizing resource allocation (El Arab and Al Moosa 2025), equitable access must remain a primary consideration (McCoy et al. 2025). To achieve these efficiencies, RAG systems must be designed with meticulous attention to detail, specifically optimizing retriever-generator interactions and hyperparameter tuning to minimize technical overhead and architectural complexity (Zhao et al. 2024a, b).

In summary, confirming the correctness and translatability of AI-prioritized variants requires multi-tiered validation and continual monitoring. Models must be benchmarked and tested against high-quality, expert-curated datasets (e.g., ClinVar or specific disease cohorts) to ensure high sensitivity (> 90%) in real-world scenarios (https://www.nostos-Genomics.com 2025a), and predictions should be verified through orthogonal biological tests. Potential orthogonal evidence-based methods include segregation analysis (Kim et al. 2019), confirming variant tracks with phenotypes in a family, and *in vivo* or *in vitro* functional assays (Kim et al. 2019; Agaoglu et al. 2022), providing experimental evidence supporting variant-mediated damage to a gene product. AI tools should follow a full product lifecycle approach, including international predetermined change control plans (PCCPs) for ML-enabled medical devices (Center for Devices and Radiological Health 2025b), with real-world performance tracked for safety and efficacy. As models evolve, outputs may change and even contradict earlier reports; this should be expected and documented so that clinicians and patients can modify care as needed (Center for Devices and Radiological Health 2025b).

## Conclusion

Incorporating ML and AI into variant analysis can transform and expedite the genetic testing process with actionable clinical intelligence, enabling earlier diagnostics and potentially life-saving interventions. When designed with transparency and community engagement, these tools accelerate variant interpretation without compromising clinical judgment or patient trust. By prioritizing ethical design, high-quality data, and explainable models, AI-assisted genomics advances the principle of beneficence by improving accuracy and efficiency, ultimately improving long-term patient outcomes.

## Data Availability

No datasets were generated or analysed during the current study.
